# The Action of Anæsthetics

**Published:** 1878-11

**Authors:** 


### ARTICLE VI.

### *The Action of Anæsthetics.*

The double danger of anæsthetics, arising from their variable influence on the respiratory and cardiac centers, has long been familiar to those engaged in their administration. This double action has lately been studied on animals by M. Vulpian, who has communicated to the Academie des Sciences a note on the subject of some practical interest. The special point which it embraces is the effect of the various anæsthetic agents on the pneumogastric, as shown by the test afforded by the experiments of Weber and Traube on the section and stimulation of the nerve.

Curara, as is well known, does not influence, in any considerable degree, the effect on the heart of faradisation of the divided pneumogastrics. The heart is arrested in paralytic relaxation, but gradually, after some seconds, its movements recommence, even though the ends of the pneumogastrics are still being stimulated. Nevertheless, the effect of faradisation of the peripheral segments of the pneumogastrics is not absolutely the same in a curarised and in an unpoisoned animal. The effect of faradisation of a single vagus is less marked, and the diastolic arrest of the heart is less prolonged under the influence of curara. If the influence of the poison is profound, there is a period during which the strongest faradisation of the pneumogastrics has no other effect on the heart than to accelerate its movements. From this it is evident that the curara does not, as was once supposed, leave intact the cardiac extremities of these nerves.

Anæsthetics, however, have been found by M. Vulpian also to have a marked and very different influence on the excitation of the peripheral extremities of the pneumogastrics, and a still greater influence on the effect of excitation of the central extremities of the same nerves. The effect of chloral hydrate may be taken as an instance of this. If a solution of chloral be injected into the vein of a dog in a quantity sufficient to produce a profound sleep, complete anæsthesia is produced. The movements of the heart and of respiration continue. Occasionally, especially if the injection has been rapid, the respiratory movements suddenly cease, the heart continuing to beat for some minutes. Commonly the breathing recommences if artificial respiration is maintained for a few minutes, or if the trunk be faradised, intermittently, about twenty times a minute. It is sometimes necessary to continue this respiration for ten or twenty minutes before the spontaneous movements recommence. Occasionally this respiratory syncope, as it is termed by M. Vulpian, occurs only some time after the injection, and during an experiment, perhaps in consequence of the traumatic irritation. Very similar accidents may be observed in animals placed under the influence of ether, chloroform and analogous substances.

Another accident which may occur in dogs under the influence of chloral, is the more or less sudden arrest of the heart's action, either during the intravenous injection or during an experiment involving irritation of the sensory nerves. The respiratory movements continue for some seconds after the heart ceases to beat. It is very rarely that the cardiac contraction can be restored, by faradisation employed at the moment at which the heart has ceased to beat. This cardiac syncope is also observed in animals under the influence of ether and chloroform. It is certainly sometimes due to the reflex influence of nerves irritated during an operation, but occurs as a result of this irritation much more readily in animals under the influence of an anæsthetic than in those which are not, or which are under the influence of curara. The comparative immunity from this accident which is presented by curarised animals is no doubt to be attributed to the influence of the poison in moderating the action of the pneumogastrics upon the heart.

From the phenomena mentioned above, it is evident that the respiratory center suffers remarkably in animals under the influence of anæsthetics, and especially under that of chloral. A slight increase in the quantity of chloral in the circulation, or a reflex influence, may arrest its action. So also with the cardiac center. If the experiment of faradising the pneumogastrics is repeated upon animals under the influence of chloral, it is found that the stimulation of the central ends of the divided nerves arrests the movements of respiration, just as in an animal of the same kind under normal conditions ; but whereas in the latter the respiratory movements go on again spontaneously and easily in most cases, in spite of the continuance of the stimulation ; they do not return spontaneously in dogs under the influence of chloral, and the animals die unless the faradisation is stopped and artificial respiration employed, either alone or with the addition of intermitting faradisation of the trunk. Sometimes a few seconds' faradisation of the superior segments of the vagus nerve is sufficient to produce this arrest. Thus under these conditions we may have, on faradisation of the central ends of the divided pneumogastrics, the same effect which M. Paul Bert has observed in animals not chloralized —sudden death. If the experiment is repeated in the same dog, the same result may be obtained two or three times, but no more. It is subsequently impossible thus to cause a persistent arrest of respiration. The spontaneous movements return after a suspension of a greater or less duration, although the faradisation of the superior extremities of the vagi is continued. If in the same complete chloralization the peripheral extremities of the pneumogastrics are faradised, the heart is arrested in diastole just as in animals, under normal conditions, and, what is rarely observed otherwise, it may be permanently arrested if the stimulation is prolonged for a short time.

These experiments illustrate very strikingly the phenomena which are sometimes observed in man, and they are of especial value in their proof of the influence which traumatic irritation may have in arresting the action of a centre depressed by the influence of an anæsthetic. But the experiments stop just where we should like them to go on. The differences in this respect, if any, which are to be observed between ether and chloroform is a point of great practical importance, and on which we hope M. Vulpian may be able to furnish us with further information.—*London Lancet.*

